# Editorial

**DOI:** 10.1017/gmb.2020.1

**Published:** 2020-08-28

**Authors:** Kristin Verbeke

**Affiliations:** Translational Research Center for Gastrointestinal Disorders (TARGID) KU Leuven, Belgium

Despite the plethora of academic journals that publish research on the microbiome, there remains a need for a specialty journal in which high-quality articles with a core focus on the *human gut microbiome* are published. This is the objective of launching *Gut Microbiome,* a new journal co-published by Cambridge University Press and The Nutrition Society.

Launching and editing a new journal is an ambitious task, which I am delighted to undertake. I am honoured to be working with an editorial board comprising dynamic and enthusiastic researchers with a broad range of expertise within the different aspects of the human gut microbiome.

For a long time, the human large intestine has been considered as an organ of little importance that merely served to prepare undigested food for defecation. However, the interest in the gut and particularly in the inhabiting microbiota has exponentially increased with the development of advanced high-throughput methodology including the different “omics” techniques that allowed detailed analysis of the microbiome. Over the past decades, we have appraised the microbiome as an intriguing and mysterious ecosystem. The possibilities to target and modify the microbiome have markedly raised expectations for preventing and treating numerous diseases. To deliver on these expectations, robust evidence and mechanistic knowledge are paramount to widen our understanding of the interactions between the microbiota and the host. It is, therefore, our belief that a journal specifically dedicated to the human gut microbiome and the complex microbiota–host interactions is timely and highly warranted.

*Gut Microbiome* differentiates itself from other journals in the microbiome field in multiple ways. First, it is unique in placing the contributing factors that influence the human gut microbiota and in turn how the gut microbiome impacts the health, development and disease status of the whole human body under the spotlight. Second, the interaction among nutrition, microbiome and health are critical to the journal’s scope, which is reflected in the support from the Nutrition Society. As stated by the World Health Organization in 2003, a well-balanced diet is a key factor to fight conditions and chronic diseases like obesity, cardiovascular disease, diabetes, osteoporosis and even cancer. However, diet is also an important determinant of the gut microbiome and the extent to which dietary modulation of the microbiota affects those health conditions requires further investigation. Therefore, the role that different diets, nutraceuticals, prebiotics and probiotics have in shaping an individual’s microbiome composition and activity and how this is translated to health benefits are to be covered.

The strong focus of this journal on the human gut microbiome and the role of nutrition do not preclude interdisciplinarity. Our hope is that the journal will contribute to the development of an integrated and interdisciplinary understanding of the gut microbiome by combining disciplines like gastroenterology, microbiology, immunology, bioinformatics, nutrition, cancer biology, microbial ecology, endocrinology and psychobiology. Importantly, manuscripts on human microbial ecosystems other than the gut and manuscripts on the animal intestinal microbiome will be welcome insofar as they are relevant to understanding the human microbiome and its interactions with the host.

To facilitate fast and widespread distribution, all manuscripts will be published according to an open-access policy. Research output will thus be available online at no cost for the reader immediately upon acceptance of the manuscripts. In addition, copyright is not transferred to the publisher but remains with the authors, thereby allowing them to freely distribute published papers. All manuscripts will be available through the web page of the journal and through major research databases.

Our hope is to serve the interests of the gut microbiome scientists by stimulating and publishing multidisciplinary research output in the human gut microbiome field, but above all, we hope you will join us in this exciting new venture and we look forward to receiving your comments and suggestions.

